# The relationship between N-terminal prosomatostatin, all-cause and cardiovascular mortality in patients with type 2 diabetes mellitus (ZODIAC-35)

**DOI:** 10.1186/s12902-015-0009-2

**Published:** 2015-04-14

**Authors:** Peter R van Dijk, Gijs WD Landman, Larissa van Essen, Joachim Struck, Klaas H Groenier, Henk JG Bilo, Stephan JL Bakker, Nanne Kleefstra

**Affiliations:** Isala, Diabetes Centre, P.O. box 10400, 8000 G.K Zwolle, The Netherlands; Sphingotec GmbH, Hennigsdorf, Germany; Department of General Practice, University Medical Center Groningen and University of Groningen, Groningen, The Netherlands; Department of Internal Medicine, Isala, Zwolle, The Netherlands; Department of Internal Medicine, University Medical Center Groningen and University of Groningen, Groningen, The Netherlands; Langerhans Medical Research group, Zwolle, The Netherlands

**Keywords:** Type 2 diabetes mellitus, Somatostatin, N-terminal prosomatostatin, Mortality

## Abstract

**Background:**

The hormone somatostatin inhibits growth hormone release from the pituitary gland and is theoretically linked to diabetes and diabetes related complications. This study aimed to investigate the relationship between levels of the stable somatostatin precursor, N-terminal prosomatostatin (NT-proSST), with mortality in type 2 diabetes (T2DM) patients.

**Methods:**

In 1,326 T2DM outpatients, participating in this ZODIAC prospective cohort study, Cox proportional hazards models were used to investigate the independent relationship between plasma NT-proSST concentrations with all-cause and cardiovascular mortality.

**Results:**

Median concentration of NT-proSST was 592 [IQR 450–783] pmol/L. During follow-up for 6 [3–10] years, 413 (31%) patients died, of which 176 deaths (43%) were attributable to cardiovascular causes. The age and sex adjusted hazard ratios (HRs) for all-cause and cardiovascular mortality were 1.48 (95%CI 1.14 - 1.93) and 2.21 (95%CI 1.49 - 3.28). However, after further adjustment for cardiovascular risk factors there was no independent association of log NT-proSST with mortality, which was almost entirely attributable to adjustment for serum creatinine. There were no significant differences in Harrell’s C statistics to predict mortality for the models with and without NT-proSST: both 0.79 (95%CI 0.77 – 0.82) and 0.81 (95%CI 0.77 – 0.84).

**Conclusions:**

NT-proSST is unsuitable as a biomarker for cardiovascular and all-cause mortality in stable outpatients with T2DM.

## Background

The hormone somatostatin plays a central role in the inhibition of growth hormone (GH) release from the pituitary gland [1]. Somatostatin is also secreted by gastric and pancreatic D-cells in response to meal ingestion through stimulation of the autonomic nervous system [2-4]. It suppresses the release of insulin-like growth factor-1 (IGF-1), vasoactive intestinal polypeptide, gastrin, secretin, and pancreatic polypeptides and exerts a range of physiological effects, such as modifying intestinal transit time and regulating intestinal water and electrolyte transport [1,5-8].

Somatostatin influences glucose metabolism by inhibiting insulin, glucagon secretion and IGF-1 production [9]. In type 2 diabetes mellitus (T2DM), somatostatin lowers glucose concentrations by inhibiting glucagon secretion [9,10] and abnormalities in the GH-IGF-1 axis have been associated with increased cardiovascular risk in T2DM [11-13].

The half-life of somatostatin in plasma is only 1–3 minutes and concentrations are generally in sub-picomolar amounts [14,15]. The stable precursor, the N-terminal fragment prostomatostatin 1–64 (NT-proSST), is secreted in the circulation along with somatostatin, circulates in approximately 1000-fold higher plasma concentrations and is considered to reflect somatostatin concentrations [16-18]. Recently, plasma NT-proSST concentrations were identified as a potential marker for acute heart failure and mortality in patients presented to an emergency department [19]. However, the long-term predictive capabilities of stable plasma NT-proSST concentrations in stable outpatients with T2DM have not been studied.

Aim of the present study was to evaluate the association and predictive capabilities of baseline plasma NT-proSST concentrations and all-cause and cardiovascular mortality in a prospective T2DM cohort.

## Methods

### Study group

The Zwolle Outpatient Diabetes Project Integrating Available Care (ZODIAC) study was initiated in 1998, in the Zwolle region of the Netherlands. One of the initial study goals was studying the effects of task delegation from physicians to specialist nurses, details have been published previously [20,21]. As a subcategory of the ZODIAC study the effects of several biomarkers, including NT-proSST, on risk prediction in T2DM were planned and blood was stored for this purpose. The ZODIAC study cohort consisted of Dutch T2DM patients treated exclusively in primary care. Patients were only excluded if they were already treated in secondary care for their diabetes, if they had a very short life expectancy (including patients with active cancer) or if they were considered to have insufficient cognitive abilities [20]. In the first year, 1,143 patients with T2DM were included, and in 2001, 546 patients with T2DM were enrolled, which resulted in a combined study population of 1,689 patients [22]. The ZODIAC study was approved by the local medical ethics committee (Isala, Zwolle), and all patients gave informed consent.

### Data collection and measurements

Baseline data were collected in 1998 and 2001, including a full medical history. The diagnosis of diabetes was based on the diagnostic criteria used in the primary care diabetes treatment guideline of the Dutch college of general practicioners of 1989 and 1999 (based on the 1985 World Health Organisation (WHO) and 1997 American Diabetes Association (ADA) criteria, respectively) [23,24]. The validity of the diagnosis type 2 diabetes was checked in the individual patient files by looking up the glucose measurements the diagnosis was based upon, and comparing these measurements with the criteria for diabetes in the national guideline for each patient [21]. Patients were considered to have a history of CVD if they had a history of angina pectoris, myocardial infarction, percutaneous transluminal coronary angioplasty, coronary artery bypass grafting, stroke or transient ischemic attack. Laboratory and physical assessment data were collected annually and included non-fasting lipid profile, glycated hemoglobin (HbA_1c_), serum creatinine (sCr), albumin-to-creatinine ratio (ACR) in a portion of urine, and blood pressure. SCr was measured by a kinetic colorimetric Jaffe method (Modular P Analyzer, Roche Almere, the Netherlands), ACR was measured using immunonephelometry (Behring Nephelometer; Mannheim, Germany), and blood pressure was measured twice with a Welch Allyn sphygmomanometer in the supine position after at least 5 minutes of rest.

NT-proSST was measured in non-fasting plasma samples collected at baseline and kept frozen at −80° Celsius until analysis in 2010. NT-proSST was measured using an assay in the chemiluminescence/coated tube-format (B.R.A.H.M.S. GmbH, Hennisdorf/Berlin, Germany) [25]. For this study, the assay used had a detection limit of 4 pmol/L; the inter-laboratory coefficient of variation (CV) was 20% at 18 pmol/l, 10% at 50 pmol/l, and <6% for plasma NT-proSST concentrations above 100 pmol/L (highest calibrator concentration used was 2500 pmol/L). The stability of the native analyte at 22°C and 37°C was tested in EDTA-plasma from 10 different individuals. At 22°C the analyte was stable (<10% loss of immunoreactivity) for 72 h and at 37°C for 24 h. Samples were analysed in duplicate. And although the samples were only thawed for analysis, prolonged frozen storage and repeated (4 times) freeze-thaw cycles had no effect on measured plasma NT-proSST concentration: in 5 EDTA-plasma samples, freezing and thawing 4 times had no influence on the measured concentration of proSST (mean values, 99.1% [range, 93.8%-104.3%] of the original values).

Baseline plasma NT-proSST values could be measured in 1,327 (79%) patients. One patient was excluded due to extreme high values for NT-proSST (71,300 pmol/L). Because not all patients had NT-proSST values, we compared the baseline characteristics of subjects from whom samples were available to those without. Besides a higher but non-relevant difference in serum creatinine among patients without NT-proSST measurements (92 [IQR 82–104] μmol/L versus 93 [IQR 84–106] μmol/L, p = 0.02) there were no significant baseline differences in patients with and without NT-proSST measurements. In a separate Cox regression analyses the association between the presence or absence of a NT-proSST measurement and CV and all-cause mortality in the combined cohort of 1,689 patients was tested. For all-cause, but not for cardiovascular, mortality there was an increased hazard ratio (HR) (1.3, 95% CI 1.09 – 1.63) for patients with missing NT-proSST measurements as compared to patients with NT-proSST measurements.

### Outcomes

Primary end-points were cardiovascular and all-cause mortality. In 2009, vital status and cause of death were retrieved from records maintained by the hospital and general practitioners. Causes of death were coded according to the International Classification of Diseases, 9th revision (ICD-9). Cardiovascular death was defined as death in which the principal cause of death was cardiovascular in nature, using ICD-9 codes 390–459 [26-28].

### Statistical analysis

Cox regression analyses were used to analyze the risk of all-cause and cardiovascular mortality during follow-up. Plasma NT-proSST concentrations were non-normally distributed and logarithmic (log) transformation was applied so the HR derived were expressed as an increase in risk per doubling of baseline plasma NT-proSST concentrations. Four models were used: (1) a crude model, (2) a model that included age and gender and NT-proSST, (3) a fully adjusted (duration of diabetes, smoking (yes/no), macrovascular disease (yes/no), BMI, systolic blood pressure, HbA_1c_, log SCr, cholesterol-HDL ratio, albuminuria (yes/no)) model in which NT-proSST was included and (4) a fully adjusted model in which NT-proSST was not included. The additional value of plasma NT-proSST concentrations for risk prediction of all-cause and cardiovascular mortality was assessed with Harrell’s C statistics. Calibration was investigated using the Grønnesby and Borgan test, assessing the goodness of fit [29]. Statistical analyses were performed using SPSS (IBM SPSS Statistics for Windows, Version 20.0. Armonk, NY: IBM Corp.) and STATA version 12 (Stata Corp., College Station, TX: StataCorp LP). A two-sided p < 0.05 was considered significant.

## Results

Baseline characteristics of the study population per quartile are presented in Table 1. The baseline median plasma NT-proSST concentration in 1,326 patients was 591 [IQR 450–783] pmol/L. Concentrations were significantly higher in women than in men (618 [IQR 474–803] pmol/L versus 558 [IQR 430–746] pmol/L respectively, p < 0.001).

**Table 1:** **Baseline characteristics of 1,326 patients presented as quartiles NT-proSST concentration**

	**All patients**	**Quartile 1**	**Quartile 2**	**Quartile 3**	**Quartile 4**	**P-value**
NT-proSST (pmol/L)	592 [450–783]	<450	450-590	590-780	>780	
N	1326	332	330	332	332	
Deceased (N,%)	413 (31)	73 (22)	74 (22)	115 (35)	151 (46)	
Follow-up time (years)	6 [3-10]	6 [3-10]	9 [3-10]	7 [3-10]	5 [3-10]	
Female sex (N,%)	738 (56)	155 (34)	182 (55)	198 (60)	203 (61)	<0.01
Age (years)	70 [61–76]	62 [52–72]	65 [58–72]	69 [61–77]	73 [65–78]	<0.01
Smoking (%)	19.0	13	16	13	13	0.28
History of CVD (%)	34	26	33	37	42	<0.01
Diabetes duration (years)	4 [2–9]	3.1 [2–8]	4 [2–9]	5 [2–10]	5 [2–10]	<0.01
BMI (kg/m^2^)	28 [25–32]	30 [27–33]	29 [26–32]	29 [25–32]	28 [25–30]	<0.01
SBP (mmHg)	150 [140–170]	150 [135–162]	150 [135–170]	150 [140–170]	150 [135–170]	0.29
HbA_1c_ (%)	7.0 [6.3 - 8.0]	7.1 [6.3 - 8.3]	7.0 [6.2 – 8.1]	7.0 [6.4 - 8.0]	7.0 [6.3 - 8.0]	0.83
HbA_1c_ (mmol/mol)						
Serum creatinine (μmol/L)	92 [82–104]	86 [77–94]	89 [80–100]	93 [84–105]	102 [89–199]	<0.01
Cholesterol:HDL ratio	4.8 [3.9 - 6.0]	4.8 [3.9 - 5.7]	4.8 [4.0 - 5.7]	4.7 [3.9 - 6.1]	4.8 [3.8 - 5.9]	0.68
Albuminuria present (N,%)	515 (39)	129 (39)	106 (32)	130 (39)	150 (45)	<0.01

Values are depicted as n (%), mean (SD) or median [IQR]. *Abbreviations*: *BMI* body mass index, *CVD* cardiovascular disease, *SBP* systolic blood pressure, *CI* confidence interval, *CVD* cardiovascular diseases, *HDL* high-density lipoprotein, *IQR* interquartile range, *NT-proSST* N-Terminal prosomatostatin.

**Figure 1:**
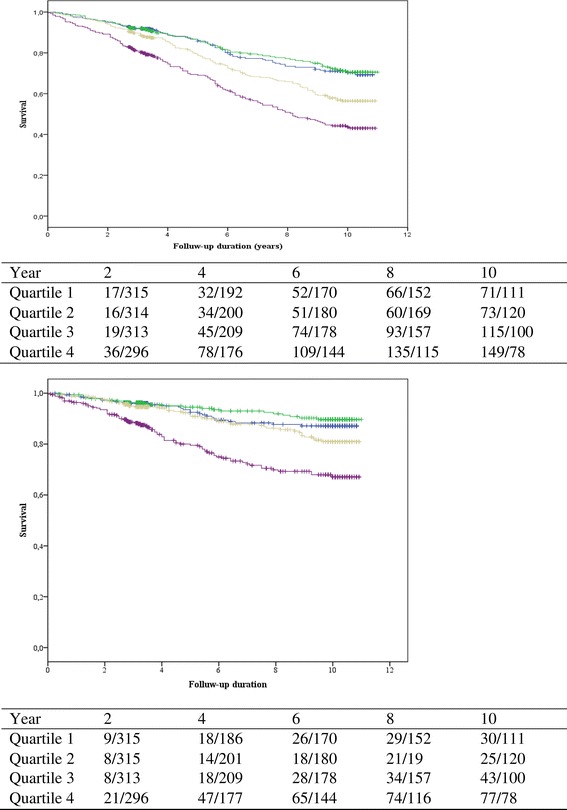
Kaplan Meier survival curves for the associations between quartiles of PSS and all-cause mortality (upper panel) and cardiovascular mortality (lower panel). The green line shows quartile 1, the blue line quartile 2, the yellow line quartile 5 and the purple line quartile 4.

During a median follow-up for 6 [IQR 3–10] years, 413 (31%) patients died, of which 176 (43%) died from cardiovascular causes. Median baseline plasma NT-proSST concentration of patients that were alive (558 [IQR 435–728] pmol/L) was significantly lower than that of patients that died (683 [IQR 514–894] pmol/L) and those that died from cardiovascular causes (743 [IQR 546–993] pmol/L) (both p < 0.001) (Figure 1).

In univariate Cox regression analyses the log NT-proSST was significantly associated with all-cause (HR 2.80, 95% CI 2.17 - 3.60) and cardiovascular mortality (HR 3.86, 95% CI 2.64 - 5.62). The corresponding age and gender adjusted HRs were 2.21 (95% CI 1.49 - 3.28) and 1.48 (95% CI 1.14 - 1.93). In the fully adjusted model, the association of log NT-proSST with all-cause and cardiovascular mortality was no longer significant (HRs 1.09 (95% CI 0.81-1.46) and 1.07 (95% CI 0.69-1.68)).

For hypothesis generation, a post-hoc, step-wise Cox model was built; the introduction of sCr to the model resulted in the disappearance of an independent relationship between log NT-proSST and all-cause and cardiovascular mortality. Furthermore, the fully adjusted model for all-cause mortality without sCr had a significantly lower goodness of fit, X^2^ 374 (*df* 11) versus 391 (*df* 12) (p < 0.001), as compared to the complete model.

The Harrell’s C statistics in Table 2 show that the more potential confounders and cardiovascular risk factors adjusted for, the better the model predicted cardiovascular and all-cause mortality. Harrell’s C-values were not different for models 3 and 4, both for all cause and cardiovascular mortality, indicating that plasma NT-proSST concentrations have no additional value on top of well-known risk factors. The Grønnesby and Borgan p-values in Table 2 indicate that, except for model 2 predicting cardiovascular mortality, all models were well calibrated. The Schoenfeld residuals showed no substantial deviations, supporting the assumption for proportional hazards.

**Table 2:** **Hazard ratio’s and additional value of baseline log**_**2**_ **NT-proSST concentrations in risk prediction compared to established cardiovascular risk markers**

	**Model 1**	**Model 2**	**Model 3**	**Model 4**
**All-cause mortality**				
Hazard ratio (95%CI)	2.80 (2.17-3.60)	1.48 (1.14-1.93)	1.09 (0.81-1.46)	NA
Harrel’s C (95% CI)	0.62 (0.59-0.65)	0.77 (0.75-0.80)	0.79 (0.77-0.82)	0.79 (0.77-0.82)
Grønnesby and Borgan test p-value	0.60	0.11	0.51	0.32
**Cardiovascular mortality**				
Hazard ratio (95%CI)	3.86 (2.64-5.62)	2.21 (1.49-3.28)	1.07 (0.69-1.68)	NA
Harrel’s C (95% CI)	0.65 (0.60-0.70)	0.76 (0.72-0.80)	0.81 (0.77-0.84)	0.81 (0.77-0.84)
Grønnesby and Borgan test p-value	0.16	0.03	0.06	0.06

Model 1: crude.

Model 2: as model 1 and also adjusted for age and sex.

Model 3: as model 2 and also adjusted for duration of diabetes, smoking (yes/no), macrovascular disease (yes/no), BMI, SBP, HbA_1c_, log sCr, cholesterol-HDL ratio, albuminuria (yes/no) and NT-proSST.

Model 4: model 3 without NT-proSST.

*Abbreviations*: *BMI* body mass index, *CI* confidence interval, *HDL* high-density lipoprotein, *HR* Hazard ratio, *SBP* systolic blood pressure, *sCr* serum creatinine.

## Discussion

This is the first study to investigate the relation between plasma NT-proSST concentrations and mortality in outpatients with T2DM after long-term follow-up. The age- and gender corrected plasma NT-proSST concentrations were associated with all-cause and cardiovascular mortality. After adjustment for all classic risk factors, plasma NT-proSST concentrations were not associated with all-cause and cardiovascular mortality and had no added benefit with regard to risk prediction.

Adding plasma NT-proSST concentrations to a model with potential confounders and well-known cardiovascular risk factors for mortality did not improve the Harrel’s C statistic compared to the fully adjusted model without plasma NT-proSST concentrations, indicating a lack of benefit in risk prediction when adding NT-proSST. The absence of a relationship is most likely caused by a true lack of additional predictive capabilities of plasma NT-proSST concentrations, although we cannot exclude that the absence of a relationship was caused by the relatively high initial predictive capability for the model without plasma NT-proSST concentrations or mutual correlations between plasma NT-proSST concentrations with 9 additional traditional cardiovascular risk markers. To explore this in more detail, we performed a post-hoc step-wise Cox model analysis in which the independent relationship between plasma NT-proSST concentrations and mortality disappeared after introduction of sCr. Since somatostatin is known to influence renal function and the administration of somatostatin analogues inhibit the GH-IGF-1 related decline in renal function in T2DM, increased plasma NT-proSST concentrations may reflect a compensatory increase in somatostatin in order to prevent progression of renal decline among patients with T2DM [30-32]. Alternatively, a decreased renal clearance of NT-proSST from the circulation could also be hypothesized in this T2DM population. Whatever putative benefit an increase in plasma NT-proSST concentrations might provide for renoprotection, there was no accompanying mortality benefit.

Some other limitations should be mentioned. Due to the exclusion of 21% patients with missing plasma NT-proSST concentrations, selection bias could have been introduced. For this reason, we calculated the HR for missing values on NT-proSST for total mortality (HR 1.33, 95% CI 1.08–1.63, in a crude model), an outcome that suggests an underestimation of the relationship observed. Furthermore, we cannot exclude the presence of difference in outcomes between patients who received task delegation care during the first 3 years of the ZODIAC study and patients who did not. Unfortunately, data on relevant comorbidity besides cardiovascular diseases are unknown. As plasma NT-proSST concentration was only measured once, correction for potential fluctuations in NT-proSST concentrations, in particular related to food intake, was not possible [3].

Nevertheless, the present study adds to the current literature by describing for the first time the predictive capabilities of NT-proSST in a large cohort of patients with T2DM with sufficient follow-up. Based on these results, NT-proSST appears to be no suitable biomarker for cardiovascular an all-cause mortality prediction in patients with T2DM. However, these results do not exclude a role for NT-proSST a potential marker for short term risk prediction and further research should focus on the use of NT-proSST as a biomarker in specific areas such as acute heart failure in non-DM subjects and several neuro-endocrine and gastro-intestinal processes [3,19,33,34].

## Conclusion

After correction for a set of well-known risk factors, high plasma NT-proSST concentrations were not independently associated with increased all-cause and cardiovascular mortality in patients with T2DM. The plasma NT-proSST concentration does not appear to be suitable as a biomarker for the prediction of mortality in stable outpatient with T2DM.

## Abbreviations

BMI: Body mass index
CI: Confidence interval
CVD: Cardiovascular disease(s)
EDTA: Ethylenediaminetetraacetic acid
GH: Growth hormone
HDL: High-density lipoprotein
HR: Hazard ratio(s)
IGF-1: Insulin-like growth factor-1
IQR: Interquartile range
NT-proSST: N-terminal prosomatostatin
SBP: Systolic blood pressure
SD: Standard deviation
sCr: Serum creatinine
T2DM: Type 2 diabetes mellitus
ZODIAC: Zwolle Outpatient Diabetes Project Integrating Available Care
